# Predictors of sustained use of mobile health applications: Content analysis of user perspectives from a fever management app

**DOI:** 10.1177/20552076231180418

**Published:** 2023-06-05

**Authors:** Sara Hamideh Kerdar, Moritz Gwiasda, Bettina Berger, Larisa Rathjens, Silke Schwarz, Ekkehart Jenetzky, David D Martin

**Affiliations:** 1 Faculty of Health/School of Medicine, Witten/Herdecke University, Witten, Germany; 2 Department of Child and Adolescent Psychiatry and Psychotherapy, University Medical Center of the Johannes-Gutenberg-University, Mainz, Germany; 3 Department of Pediatrics, Eberhard-Karls University Tübingen, Tübingen, Germany

**Keywords:** mHealth, mobile health apps, sustained use, user, feedback, FeverApp, register, fever‌

## Abstract

**Objectives:**

Mobile health applications could be means of educating and changing behaviours of their users. Their features and qualities determine the sustainability of use. The FeverApp with two main features of information and documentation is a research-based app. In this observational cohort study, to evaluate the influential predictors of use, users’ feedback on the FeverApp, were analyzed.

**Methods:**

Feedback is given using a structured questionnaire, four Likert items and two open questions regarding positive and negative impressions, available via app menu. Conventional content analysis (inductive approach) on the two open questions was performed. Comments were grouped into 12 codes. These codes were grouped hierarchically in an iterative process into nine subcategories and lastly into two main categories ‘format’ and ‘content’. Descriptive and quantitative analysis were performed.

**Results:**

Out of 8243 users, 1804 of them answered the feedback questionnaire. The features of the app (*N*  =  344), followed by the information aspect (*N*  =  330) were most frequently mentioned. Documentation process (*N*  =  226), request for new features or improvement of the current ones (*N*  =  193), and functioning (*N*  =  132) were also highlighted in users’ feedback. App's ease of use, design and being informative were important for the users. The first impression of the app seems important as the majority of feedback were given during the first month of using the app.

**Conclusion:**

In-app feedback function could highlight shortcomings and strengths of mobile health apps. Taking users’ feedback into consideration could increase the chance of sustained use. Besides ease of use and clear, likeable designs, users want apps that serve their needs while saving time.

## Introduction

Mobile health (mHealth) apps are recognized as valuable sources of connection between healthcare practitioners and the public.^1^ Many mHealth apps have been developed in order to help individuals to be encouraged and independent regarding their health and as a result with the intention to reduce the costs of healthcare services.^2^ These apps provide different opportunities and have different objectives such as distribution of information, collection of data, monitoring patients,^1^ and supporting patients with chronical diseases and their medication adherence.^3^ To reach these objectives, many mHealth apps in various medical fields such as mental health or diabetes for different age groups have been developed. Additionally, mHealth apps provide a unique opportunity for researchers to collect data using different methods such as self-report data, ecological momentary assessment (EMA),^4^ or ‘citizen-led’ data (i.e., data collected from the general public)^5^ with considerable number of participants, targeting different groups.^6^ Moreover, mHealth apps can be sources of education for patients and caregivers by providing easy access to platforms.^3^ Through different interventions such as gamifications, personal reminders, or feedback functions, mHealth apps suggest a promising future of behaviour change.^3^ However, the efficacy of such tools remains unclear. For example, in a systematic review and meta-analysis, Wu et al.^7^ concluded that mHealth apps for diabetes are impactful tools for type 2 diabetes but inconclusive for other types. Therefore it is important to understand under which conditions users perceive such tools as helpful and have sustainable use.

In the field of paediatric fever management, studies show that parents experience high anxiety about paediatric fever and take unnecessary measures.^8–10^ Researchers have implemented different types of education for caregivers to reduce this anxiety. However, it seems that caregivers prefer to have multimedia type of information, including videos, text, images, etc.^11^ In this regard, in 2019, a research project, the FeverApp registry, was funded by the Federal Ministry of Education and Research (BMBF) in Germany.^12^ A mobile application, the FeverApp, is a means of data collection for this registry.^4^ The FeverApp registry intends to (a) increase the knowledge regarding parental fever management at home, (b) evaluate guidelines adherence, (c) enhance parental knowledge and confidence in dealing with paediatric fever, and (d) reduce the unnecessary medication intake and unnecessary visits to doctor practices.^13^

One major concern about mHealth apps is the sustainability of use as the users often leave or forget them.^14,15^ The sustainability in the use of these apps is not only one of the key elements of change in behaviour^14^ but also an important element for research studies using mHealth apps.^6^ If long-term use of these apps is wanted, users’ reviews and comments about the apps are valuable sources of information. Such feedbacks provide users’ experiences and highlight the factors that would help users and lead them to sustainable use.^16^ Although several studies have analyzed users’ public reviews regarding mHealth apps in specific themes (e.g., mental health,^16–18^ weight loss^19^ or diet^20^), the knowledge remains limited.^15^ Therefore, this study intends to explore the important factors from users’ perspectives based on the reviews given by the FeverApp users. Determining such information helps to identify the predictors of sustained use by mHealth app users.

## Methods

The user feedbacks are generated from the registry's data pool in a context of an observational cohort study. Therefore, the STROBE guidelines for cohort studies were followed.^21^

### FeverApp features and data collection

The FeverApp collects EMA data on febrile illnesses and consists of two main features documentation and information. In the documentation, which is in the form of questions and answers, users can document all fever episodes, symptoms, warning signs, etc. In the information section, users have access to a multimedia library regarding fever and the safe ways to handle it. Other features within the app are a graph overview of all fever episodes (i.e., user entries) and reminders (an alarm and different types of pop-up notifications). The app is currently (version 1.9.6) available in seven languages (German, English, French, Russian, Turkish, Arabic and Farsi). The feasibility and interactions within the app have been already published.^13^ The study has received a positive vote from the ethics committee of the Witten/Herdecke University on pseudonymized data collection (#139/2018).

The FeverApp is introduced by paediatricians and family doctors to parents. The participating doctors receive a unique practice code. The app is accessible using this code that will be used upon onboarding and will generate a family code automatically. Family members can use the family code to share created profiles and entries. Although the app was initially designed for parents and their children, any individual can use it as a documentation and education tool regarding fever and febrile illnesses. User entries (i.e., data) are stored on the servers of the University of Witten/Herdecke in Germany. Users receive the initial explanation regarding the app and the research project either directly by their doctors or via a flyer (which they receive in their doctor's practice). After the app's installation and before profile creation (onboarding) users are once again notified about their participation in a research project. Users must electronically provide informed consent to participate in the study and are informed about data privacy and data collection (i.e., where and how their data are saved) before they can use the app and create their profiles. Before onboarding, users are also notified that participation in the study is voluntary and they can withdraw at any time and delete all their data. For analysis the data can be extracted in CSV format on demand and are processed in SPSS V26 (IBM Corp., Armonk, NY, USA).

Within the app, users can give feedback by completing a feedback questionnaire, accessible in the app's menu. This questionnaire consists of four rating questions on a Likert Scale, 1 being very bad/not at all and 5 being very good/very much. The questionnaire finishes with two open questions of ‘What did you like about the FeverApp?’ (Question 5) and ‘From your point of view, what could be (even) better about the FeverApp?’ (Question 6). Data collection (i.e., the research project) was started from September 2019 and continues till the time of this publication. The data used for this study is from September 2019 until November 2021. The app functions offline, although for synchronization between different devices an internet connection is needed. It is noteworthy to mention that no trial period is defined for the users, which means they can access the app and its features as frequently and as long as they would like. The app collects EMA data, thus no follow-up studies are conducted. As the knowledge of the app features help to understand the content analysis of the user feedbacks, Table 1 briefly explains its main features.

**Table 1.** FeverApp main features.

FeverApp features	Explanations
Documentation	A questionnaire containing questions about temperature, well-being, symptoms, warning signs, Covid-19, medication, doctor visit, prescription, etc. (Figure 1(a))
Tour	Users are introduced step by step into the app's functionality. In first entry after downloading the app, users receive tips while filling the questionnaire based on their answers (Figure 1(b)).
Summary	At the end of each entry, users receive tips and feedback based on their entries.
Graph	An overview of phases of fever, based on the user's documentation. (Figure 1(c))
Info Library	23 chapters of information regarding paediatric fever and ways to handle it. It consists of text, pictures and videos. (Figure 1(d))
Reminders	1) Alarm, 2) Pop-up notifications when users do not give a new entry (a) more than 24 h, (b) after 48 h and each three months of installation.
Medication scanning	Upon scanning medications’ barcodes some fields are automatically filled.
Family synchronization	Families can use a single code to share the same children's profile and update it parallel.

**Figure 1.**
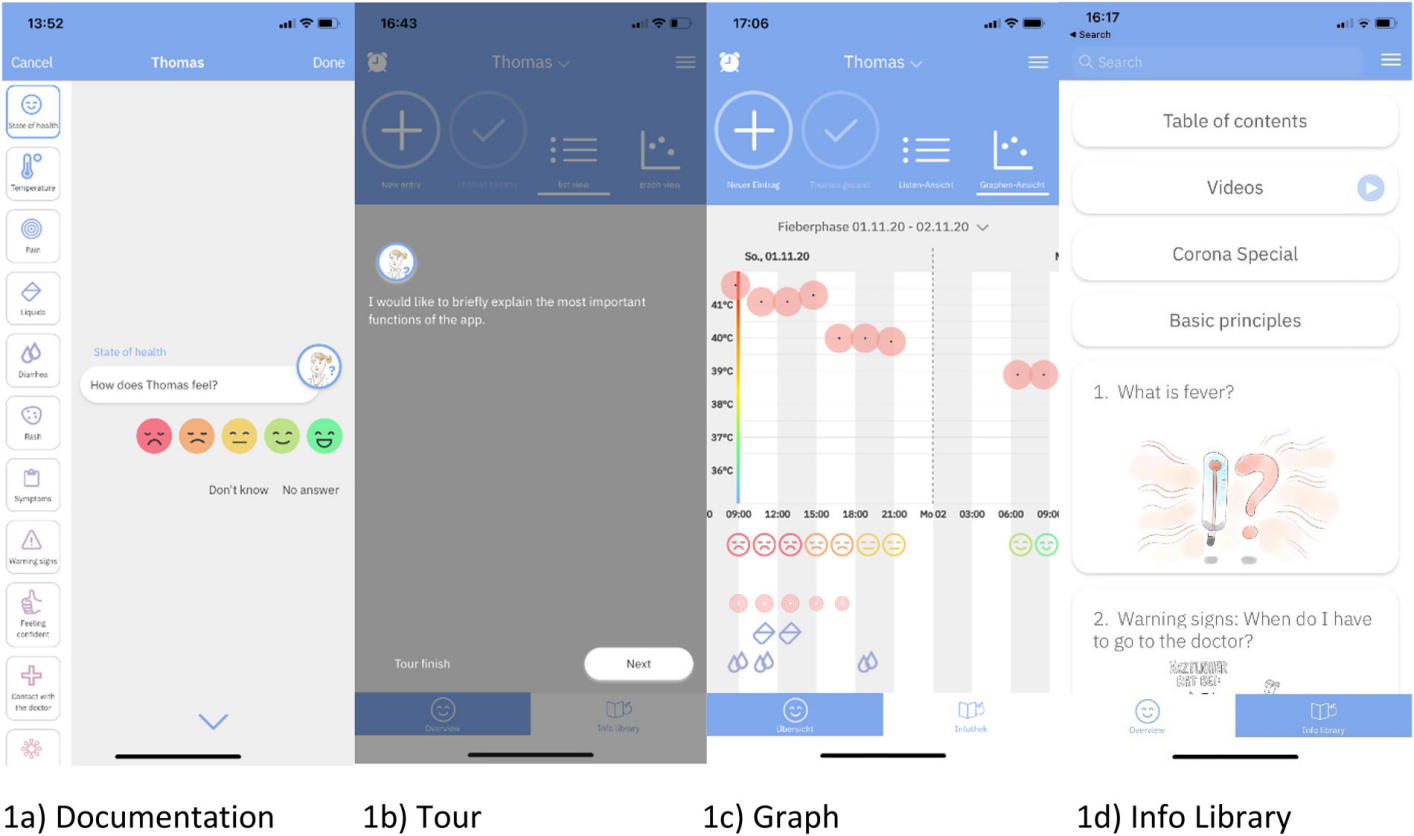
Screenshots from the FeverApp features. (a) Documentation, (b) Tour, (c) Graph and (d) Info Library.

### Conventional content analysis

To the best of our knowledge, no other studies have focused on users’ perspectives regarding educational and research-oriented mHealth apps to support parents in managing the fever of their children. Considering the limited literature for such analysis, a conventional content analysis (i.e., an inductive approach) on the open questions of feedback questionnaire (i.e., Questions 5 & 6) was performed. Inductive approach as a method of qualitative analysis is recommended, in cases where knowledge is inconsistent or absent.^22^ Accordingly, the codes and categories were shaped by the data as no previous study regarding educational and research-oriented mHealth apps was found in the literature. Therefore, the following steps recommended by Hsieh and Shannon^23^ for conventional content analysis was taken:

*Code generation*. The first author (SHK) initiated the initial reading of the comments thoroughly and repeatedly, in order to get familiarize with their contents. In this step, all comments in the seven available languages of the app were considered for analysis. However, the comments that were not understandable, very vague or too general (exclusion criteria) were disregarded (*N*  =  16). For example, comments with ‘everything is good’, ‘everything’ or ‘nothing’ were not considered. Next, the comments were read word by word by two authors (SHK & MG) and were coded based on their content to generate initial codes. Each comment was coded minimum once, but they could be coded to more than one code as well. Initially, 33 codes were recognized in the comments. The authors (SHK, MG, BB, LR) met several times to find the connection and similarities of these codes in order to define the final codes. Regular meetings among the others were a helpful way of reducing any chance of bias in interpreting user comments. Based on the similarities of their content and meaning, 12 codes were finalized and defined. All the comments were written in either German or English (no comments in other languages were given at the time of this publication). In this paper, German comments are translated into English. The comments (i.e., texts) had an average of approximately 20 words for improvement comments (Question 6) and approximately 9 words for positive comments (Question 5).

*Subcategories*. The assigned codes were grouped (by SHK) and hierarchically ordered in an iterative process and combined into subcategories (*N*  =  9). As the majority of the comments were in German, a native speaker (MG) read all the comments once again and when adjustment was needed, comments were assigned to other categories based on their content. It is noteworthy that different codes were assigned to a single category. The codes also became categories if the central superordinate aspect of a group of codes has already been grasped quite precisely beforehand.

*Main categories*. Two main categories, reflecting the essence of the subcategories were further detected.

### Quantitative analysis

For further analysis of the factors related to the users’ comments, the frequency of codes was calculated. Furthermore, the sociodemographic information of the FeverApp users was extracted from the data. Chi^2^ analysis was used to understand the association of reviews with sociodemographic, usage of app (user engagement), association between the first download to giving feedback.

## Results

### Participants

At the time of this study, the FeverApp had 8243 users, out of which 1804 of them answered the feedback questionnaire. From those who answered the questionnaire, 812 of them answered the open questions (Questions 5 and 6). Table 2 summarizes the demographic information of FeverApp users with a comparison of those who provided feedback versus those who have not provided any feedback. Several users (*N*  =  17) answered the questionnaire twice on different dates.

**Table 2.** Demographic information of FeverApp users.

	Users who gave feedback *N* = 1804 (22%)	Users who have not given feedback *N* = 6439 (78%)	*p*-Value (Chi^2^-Value)^b^
Demographics	*N* (%)	*N* (%)	
**Role**			
Mother	1574 (87.3)	4977 (77.3)	<0.001 (93.56)
Father	187 (10.4)	1266 (19.7)	
Others	6 (0.3)	67 (1.0)	
Missing	37 (2.1)	129 (2)	
**Age** (year)^a^			
14–20	6 (0.3)	84 (1.3)	0.007 (84.42)
21–30	336 (18.6)	1218 (18.9)	
31–40	1216 (67.4)	3917 (60.8)	
41–50	207 (11.5)	731 (11.4)	
≥51	8 (0.4)	91 (1.4)	
Missing	31 (1.7)	398 (6.2)	
**Education**			
Highest (Abitur)	958 (53.1)	2964 (46.0)	<0.001 (24.84)
High (Fachhochschulreife)	364 (20.2)	1220 (18.9)	
Moderate (Mittlere Reife)	363 (20.1)	1243 (19.3)	
Low (Hauptschule)	73 (4.0)	405 (6.3)	
No education	12 (0.7)	73 (1.1)	
Missing	34 (1.9)	536 (8.3)	
**Nationality**			
German	1557 (86.3)	5212 (80.9)	0.155 (100.33)
Austrian	15 (0.8)	53 (0.8)	
Polish	18 (1.0)	72 (1.1)	
Turkish	22 (1.2)	67 (1.0)	
Swiss	7 (0.4)	29 (0.5)	
Others	74 (7.2)	423 (6.56)	
Missing	111 (6.1)	583 (9.1)	
**Residency**			
Germany	1645 (92.1)	5592 (86.8)	0.177 (15.11)
Austria	9 (0.5)	36 (0.6)	
Switzerland	6 (0.3)	46 (0.7)	
Others	3 (0.3)	9 (0.7)	
Missing	142 (7.9)	756 (11.7)	
**Device**			
Android	992 (55.0)	3365 (52.3)	<0.001 (104.90)
iOS	806 (44.7)	2668 (41.4)	
Missing	6 (0.3)	406 (6.3)	

^a^ Age was calculated based on the year of this study (i.e., 2021).

^b^ The association between users who gave feedback vs. who did not.

### Answers to the feedback questionnaire

The first four questions of the feedback questionnaire and the descriptive analysis are summarized in Table 3. The frequencies are based on all the given ratings (*N*  =  1821), i.e., responses from users who have answered the questionnaire twice are also included. A strong tendency to positive ratings, was observed.

**Table 3.** Frequency of answers to the first four questions of the feedback questionnaire.

Question	Likert scale	N (%)	Mean (SD)/median
How do you like the FeverApp in general so far?	1 to 5 stars	1805 (98)	4.10 (0.80)/4
	1 = very bad		
	5 = very good		
How do you like the design of the FeverApp?	1 to 5 stars	1808 (99)	4.27 (0.75)/4
	1 = very bad		
	5 = very good		
How do you like the usability of the FeverApp?	1 to 5 stars	1808 (99)	4.13 (0.88)/4
	1 = very bad		
	5 = very good		
Do you have the impression that the app has increased your confidence in dealing with childhood fever?	1 to 5 finger	1788 (98)	3.83 (0.94)/4
	1 = no, not at all		
	5 = yes, very much		

### Conventional content analysis

*Code generation*. The answers (i.e., comments) to Question 5 (*N*  =  602) and Question 6 (*N*  =  574) (total of 1176 comments) were grouped into 12 codes. Table 4 presents the list of these codes, their definition, examples and their frequency (number of times codes were detected in the comments).

**Table 4.** Transformation of users’ comments to codes.

Codes	Definition	Examples^a^	Frequency N (%)
Features	Comments regarding the app features	‘Data is clearly presented in the graph.’ ‘Everything can be entered quickly and all family members have an overview’.	344 (29.2)
Information	Comments regarding the app being informative and helping users to learn	‘The summary with additional information and descriptions at the end of the questionnaire. The additional possible information in the app is also very useful’. ‘Exact questions train your own perception’.	330 (28.0)
Input	Comments regarding documentation process	‘It would be good if all questions do not have to be answered every time a temperature is measured. Some questions do not change every 2 h and answering them is time-consuming when you actually have to take care of your sick child’.	226 (19.2)
Requests (others)	Comments regarding features/ functions that are not available in the app. Request for further information (e.g., entry- related or age-related)	‘It should be possible to print, send and save the documentation as a pdf file. The paediatrician should be able to access the current documentation temporarily by giving his consent in the app’.	193 (16.4)
Function	Comments regarding the app's and features’ functionality	‘Setting the date and time is tedious on the smartphone because it tends to slip’.	132 (11.2)
User-friendliness	Comments that user mention when the app is/isn’t easy to use and handle	‘easy to use’. ‘The simplicity of operation and the additional explanations’ ‘Improve usability a little’	120 (10.2)
Clarity	Comments demonstrating the clarity of working with the app and clarity of its content	‘Too complicated’. ‘Clear questions and statements’.	91 (7.7)
Confidence	When the app influences user's confidence in dealing with fever	‘[The app] gives confidence in dealing with fever without giving you a false sense of security’.	49 (4.1)
Appearance	Comments regarding the app design and overview of the data	‘The design is really very nice’. ‘Question mode is quite appropriate. Children like to participate. The smileys for well-being help’.	44 (3.7)
Practicality	Comments demonstrating if the tips and information within the app and the app itself is practical	‘It is certainly practical in order to better assess the course of a disease for a doctor, or to be able to better match symptoms’. ‘I honestly don't understand the point of this app’.	23 (1.9)
Trust	Comments that demonstrate users’ trust on the app and its content	‘Son had a fever of 39.3 degrees at night, without the app I would have gone to the clinic, which would not have been necessary’.	19 (1.6)
External factors	Comments regarding time or stress restraints stopping users to use the app	‘It takes a lot of time to answer all the questions. This is difficult to organize with two children who are also sick at the same time. At the same time, the detailed query is great because you get detailed help’. ‘I would find it better if the input of the temperature would be saved independently from the other parameters. Often I don't have time to enter many details and then I have to click through’.	18 (1.5)

^a^ The comments are based on two questions of ‘what did you like about the FeverApp?’ and ‘From your point of view, what could be (even) better about the FeverApp?’.

*Sub categories and main categories*. In order to have a more clear understanding of the reviews, the generated codes (Table 4) were grouped into 9 subcategories and eventually to two main categories: (a) *Content* that contains comments regarding all the information within the app and users’ feedback about them. (b) *Format* that contains comments regarding the app features, design, ease of use, and so on. For better understanding of the categories, Table 5 summarizes subcategories and main categories alongside some examples.

**Table 5.** Main and subcategories.

Main categories (Definition)	Subcategory	Examples
Content (Comments regarding information within the app)	Trustworthiness	‘That I now know better when we need to see the doctor:’ ‘The explanations of the individual symptoms are very helpful. They give me as a mother more confidence in dealing with the fever’.
	Practicality	‘It is very informative, but a flyer would do’. ‘Gathering experience for the future, tips and tricks’.
	Informative	‘The info that followed my answers at the end was very helpful’: ‘The video is very informative. The interpretation of the temperature with recommended action is helpful’.
	Clarity	‘That you can read everything at any time and it is very clearly written! That gives you a piece of security’.
Format (Comments regarding the app features, design, usability, etc.)	Features presentation	The graph view is a bit confusing at times (colour choice and too much information when I was only trying to look at the temperature + the last time I gave the medication)’. ‘1) On the graph to link measurement's points with a line. 2) Put usage of the medicine directly on the graph, that it's clearly visible together with the fever measurements. Now it's too far down, and it's difficult to see e.g., how long was the effect’.
	Features functionality	‘That you are reminded if you have not entered anything for a long time. And the tips on how to deal with it’.
	Documentation	‘I do not fill out further measures, since I find the questionnaire altogether quite long as documentation in between and many of the further measures mentioned I find natural with a sick child to accomplish’. ‘Documentation is now easier’.
	Comprehensible	‘A clear fever log in an app’. ‘Summary with explanations and the information about fever clearly arranged’.
	Requests	‘Filling of the vaccination dates as soon as the child's date of birth has been set. Then you could also refer to it better’.

*Main categories*. The two main categories of content and format will be explained in further detail:

1. Content

*Trustworthiness*. On the one hand, users expressed their trust in the app and its content, where having the app helps them to feel more confident managing fever. For example, ‘… now I know better when we need to see the doctor’ or ‘.. helps with initial uncertainty’. Additionally, they would trust the recommendations of the app regarding when to visit a doctor. For example, ‘..That info is given whether you should go to the doctor or not’. On the other hand, some users found the app not a replacement for a doctor especially if the child is very unwell. For instance, ‘Helpful tips but also only to the extent of not replacing a visit to the doctor in an emergency’, ‘…Of course, in case of uncertainty, useful tips and advice are available in the app, but these do not replace a doctor, and I am of the opinion that it is better to go once too frequently, than to wonder later why I did not go in time’.

*Practicality*. The majority of comments on practicality of recommendations and tips were positive. For example, ‘…deepening information helps us to get further knowledge! Calf wrap! I have always felt insecure. Now I am motivated to try them out soon and to do without medication’ or ‘It is certainly practical to help a doctor to better assess the course of a disease, or to better match symptoms’. However, limited number of users did not find the app practical and reported that the app did not increase their knowledge, for example ‘I am just as smart as before and have the feeling that only data is collected here’ or ‘I did not get any really new insights, but a verbally somewhat modified presentation of my own answers’.

*Informative*. The second most common feedback was regarding the app's information function. The majority of comments expressed positive feedback. Many users found the app in general very informative. For example, ‘The tips and knowledge of why the child feels the way he does and what can be done supportively’ or ‘the app is very informative’. The comments addressed different elements of the app being informative; the Info Library, the summary, the tour and the questions. For the Info Library, all the comments were positive accompanied with requests. For example, ‘Info Library is helpful because it is informative and clearly structured’. Through the tour, the app delivers further educative information. The first time that the user answers the questions (i.e., the first use of the documentation feature) for each question category they receive a tip (this is part of the tour function of the app). For example, before the questions regarding temperature measurements, the app would recommend how parents could measure the temperature accurately. The users seem to find not only these tips but also the questions as educative. The fact that questions are so detailed helps users to observe their child more accurately. For example, they found the questions as their guide or a good way to train their observations, for example, ‘The details on what to look for when my child is sick’ or ‘That I was asked questions about the condition, for example, with the feeling of the forehead, hands and feet. That you are told what is okay and what is not, so the tips are very helpful’,

Additionally, once the users finish answering the questions and documenting their entries, the app provides a ‘summary’ of the entries with recommendations for treatment or management of fever based on the answers given. For example, by signs of dehydration, parents are advised to frequently offer their child to drink in small quantities or when would it be necessary to visit a doctor. Many users found this summary very helpful. For example, ‘The info that followed my answers at the end was very helpful’ or ‘To have certain symptoms checked and interpreted. I find this very helpful!’. Some users however would prefer to have more tips or individualized recommendations, for instance, ‘Have wished for more tips in dealing with only mild fever after for example vaccination etc’. or ‘Even more individual tips’. Several users wished that the information were based on the age of their child. For example, ‘…tailor the information more to the person (for example, a child <4 months has different requirements than older children or older people)’, ‘I would have liked more specific information and recommended measures for infants and babies’.

Although the majority of the users found the app very informative, a couple of users thought the information in the app is arbitrary and did not find the app informative enough.

*Clarity*. The users’ opinion regarding the clarity of information and recommendations were mostly positive. For example, they found ‘Clear questions and messages’ or ‘Clear structure, clear recommendation of what to do’ of the app as a positive aspect. However, some users found the information unclear, for example, ‘The clarification about the next steps is a bit confusing’ or ‘It has not been clear enough to me what exactly I should do better or differently now’.

2. Format

*Features presentation*. The overview of the app was appreciated by several users. For example, ‘The app is easy to use and gives us parents a super organized overview of the height, symptoms, etc. of the fever. In addition, I find the small Info Library super’ or ‘I think it's great to be able to document directly and get information about it. Otherwise, you would want to document somehow, but that would not be so well structured. Also the constant info, whether the temperature is still normal or elevated or is already fever, helps me a lot’. Users appreciated the way of documentation in the app and the way the questions are asked. For example, ‘Question mode is perfectly appropriate. Children like to participate. The smileys for well-being help’. Additionally, some users found the presentation of their entries in the app likeable. The general feedback was ‘Great overview of the [fever] course’. Mostly the users were happy to have their given data on the graph view. However, some users preferred to see a curve of the fever on the graph ‘The graph could be connected, so the fever curve is more visible’.

Another feature that received multiple comments was the medication feature ‘I really liked the option of selecting medications. Scanning is a significant time saver’. Despite having a scanning function to speed up the process for parents, some users found it very confusing, addressing many questions or requests accordingly. For example, ‘Simplify medication administration with daily administration’.

*Features functionality*. The comments regarding the features’ functionality mostly addressed the documentation feature. Detailed information was reported when they were troublesome or when their speed of documentation was effected, noting that with having sick children at home, one would like to document the fever episodes as fast as possible.

In the documentation functionality, most of the questions are followed by a question regarding the date and time of which the action (e.g., taking medicine, visiting doctor) took place. Some users found this question for the non-pharmaceutical actions (e.g., cuddling, singing) irrational and had difficulties documenting such information. For example, ‘The input for the taken measures is annoying, because, for example, I provide a low-stimulus environment all day or hold my child in my arms, not only at a certain time of the day’*.* Additionally, many users addressed the problem while choosing a date/time, with the scrolling function cumbersome. ‘Entering date and time of measures is cumbersome and does not work well – thus very annoying, so it is better to leave it out. It would be much faster to enter it by hand or to offer a field where you only enter it if it differs from the selected time for the entry’.

Users also reported a specific problem in the Info Library, ‘When I read through the information and click back, I don't want to start at the top of the list, but at the place where I was last’.

*Documentation*. The documentation feature of the app was valued by users as a practical and guided alternative to recording the fever phases on paper, with the extra bonus of being able to share entries and profiles with family members. They could also come back to their entries at any time and evaluate the frequency and well-being of their child through the course of the illness.

The biggest and most common comment was regarding the documentation process. Although many users appreciated the style and the questions, some users found it time-consuming and unnecessary to answer all the questions every time they document a fever episode. For instance, ‘It is very tedious to click through all the questions for each entry’. As the app recommends that parents make three entries per day when their child has fever, many parents find it unpractical to answer all the questions every time. Some parents preferred to document only the temperature and medication frequently but to avoid the whole questionnaire for each entry.

*Comprehensible*. On the one hand, the users found the app very clear and mentioned its clarity as one of its positive features. On the other hand, some users found the app confusing and had difficulties to find and use the app features. For example, even though the app has an alarm and a reminder system, some users still requested for such functions, for example, ‘Reminders (timers) for specific points in time (e.g., when taking a temperature)’. One of the most missed functionality of the app was the possibility to skip questions or chose the question category in the documentation feature. As answering all the questions every day is time-consuming, many users found it very irritating and requested ways to skip questions (even though the tour in the beginning explains the possibility). Even though the question regarding the vaccination is available, several parents wanted to explicitly document fever after a vaccination.

Additionally, despite the fact that the tour explains how to edit or delete entries, many users missed the information and commented about it. For instance, ‘Possibility of correction if one has made a mistake in the application (not possible? I did not understand how?)’

*Requests*. Many users have requested new features for the improvement of the app. The majority of the comments were about the information aspect of the app. Users requested to have age-related tips and information. Moreover, users prefer to have information related to their child's age or more specified to their answers. For example, ‘Classification into age groups (baby, toddler, child, adolescent) and for babies and toddlers who cannot yet explain the symptoms, more help to be able to better interpret symptoms in them’. Some users asked for more information in general or specific details such as how to handle a child who had behaviour changes due to their sickness. The information regarding perceived common reasons of fever, e.g., teething and vaccination^24^ was several times requested. ‘Sometimes offers few answers. E.g. When fever due to teething in babies’*.*

Users also requested to have direct contact with their paediatricians via the app or for their doctors to be able to see their entries. Additionally, they requested additional features, for example, to be able to create PDF format files from their entries and to be able to print or email them.

### Quantitative data analysis

The feedback questionnaire was answered mostly in the first month of user profile creation (*N*  =  1160, 64%), out of which 43% of them were answered on the first week of user profile creation. The users gave feedback in the second month (15%) and in the third month onwards (21%) (Range 0–25 months).

The intensity of the app usage was defined based on the use of the documentation feature. By the time of analysis, 46.2% (*N*  =  3814) of the users did not document any fever events (probably because there child had not had a febrile disease at the time). Further, 19.6% (*N*  =  1620) of the users documented once or twice (low use of documentation) and 34.2% (*N*  =  2826) of them were intensive users who have documented in the app more than two times. Intensive users provided the majority of the feedbacks (*N*  =  1455, 80%), followed by low use of documentation (*N*  =  301, 16%) and no documentation (*N*  =  65, 3.6%). The Chi^2^ analysis showed a statistically significant association between use of the app and answering the feedback questionnaire (Chi^2^ = 2354, *p* < .001).

## Discussion

Although mHealth apps are great sources of providing information or supporting patients to independently manage their diseases, an ‘active’ involvement with the apps is necessary.^25^ The withdrawal percentages from mHealth apps are a concern. One way of improving users’ experience, sustainability of use and a better chance of behaviour change, is to review and implement their feedback regarding the apps they use. Standardizing the evaluation of users’ feedbacks for all the mHealth apps is not possible, as each app offers different features for different purposes.^14^ Several studies have investigated users’ public feedback.^14–18,20^ However, despite the usefulness of such analysis in increasing the knowledge in improvements of mHealth apps, literature on this subject remains limited.^26^ There is a lack of insight regarding users’ opinions related to the mHealth apps used at the same time for research projects and targeting behaviour change. Therefore, the current study investigated users’ reviews of an educative research-based app, the FeverApp, in order to provide insight regarding the strong and weak features of such apps based on users’ reviews and to understand the qualities mHealth app users look for.

The inductive analysis in the current study showed that both the format and content of the app are important factors for users. Besides clear, likable design, users valued the app's ease of use. These findings are consistent with previous studies.^14,27^ Users found the content very informative and appreciated the different styles of information. However, several users wished for more information. The need for personalized information and recommendations has been observed in previous studies.^28^ This is also in agreement with the fact that individuals seek the internet or use self-diagnostic apps for the diagnosis of their symptoms.^29^ The FeverApp users also requested to have more age and symptom-related recommendations and information. One explanation could be that a patient always receives personalized recommendation from their doctors during a visit. Naturally, they would expect to receive such information from mHealth apps, if they are supposed to be helping the users’ self-management of health. Therefore, considering tailored information for each user could be an encouraging factor for the use of the mHealth apps.

Similar to previous studies,^14^ saving time and a fast way of using the app were mentioned in many reviews, addressing different features. For example, the scanning function of medications was appreciated as a time saver. However, the documentation format of the app that comes i a question-and-answer style received controversial feedback. Many users appreciated this style and found the questions educative and their order of appearance helpful for learning to observe their children in a thorough and structured manner. Despite this satisfaction, some complained about the fact that the app seems to suggest they must answer all questions (even though skipping questions are possible and introduced in the tour function). Considering they were dealing with an unwell child, some users wanted to document the whole questionnaire only once and then only the temperature measurements and medication intakes for the rest of the day. Even users, who wrote positive comments about the documentation feature and the questions, requested the possibility of short version of the questionnaire as well. The fact that the app provides such a possibility but that many of the users missed this prompted improvements for the next updates of the FeverApp. The fact that engagement with apps decreases when users have to enter data manually^25^ highlights the importance of a smooth style of documentation for mHealth apps for a long-term sustained use.^14^

Sheppard^2^ argues that the success of mHealth apps depends on the trust built between the users and such technology. Although privacy concerns have been identified in several studies as a factor influencing sustained usage of mHealth apps,^25,30^ the FeverApp users did not raise any privacy concerns. Several factors could influence such trust. Firstly, in the case of FeverApp, the access code is distributed to parents by their doctors and paediatricians. This is in accordance with the results of the study by Vo et al.^31^ that the support of health providers could facilitate the issues impacting the use of mHealth apps (such as privacy or information validity concerns). As the validity and medical accuracy of many of mHealth apps are under question^32^ and the fact that some apps become suddenly unavailable,^30^ trusting mHealth apps seems to be difficult for many users. Secondly, the app informs users that it is based on a research project funded by the German Ministry of Education and Research (BMBF) and the content is developed by collaborations of doctors, paediatricians, nurses, parents and psychologists. Some users found these factors positive, for example, ‘The opportunity to contribute to medicine and science’ or ‘the fact that one supports the research’. Previous studies show that individuals would like to have a trusted source such as official organizations or healthcare providers to approve the apps.^27^ Finally, in agreement with previous studies,^17^ having clear explanations about data privacy, place and ways of storing users’ data, pseudonymized data collection, and providing ways for the users to revoke their information at any time seem to be additional strength for the privacy assurance of the FeverApp users. Böhm et al. (2020) argue that not providing personal information in the optional fields could be a sign of having privacy concerns.^25^ However, the lack of answers could be associated with other aspects. Some users find providing such information unnecessary as the app purpose is to educate and document fever in children. For example, a user commented ‘… I don't understand why one has to specify one’s educational background*’*.

The possibility of leaving feedback is a great opportunity for mHealth app users to mention their requests for specific apps. Users appreciate the possibility to be in direct contact with health providers via mHealth apps.^15,28,31^ Users become encouraged when they receive a quick answer to their questions from their practitioners through the app.^33^ The FeverApp users have also requested such features, in order not only to have answers to their questions but also for their practitioners to have access to their entries. The possibility of a direct contact with doctors (or ‘designated individuals’) through mHealth apps has been mentioned as positive aspects in other studies.^14,15^ Similar to the users of mental health apps,^17^ FeverApp users wanted to share their data with their health providers by having either an export function or the possibility of emailing their data. This type of feature should be considered in mHealth app developments as it provides security, trust, and possible long use of the apps. However, such features need a broad consideration of data privacy based on each country's regulations.^34^ Additionally, mHealth app users consider sharing features as a positive benefit of the apps, sharing their data with their families or friends.^27^ Several users in the current study appreciated the family code feature of the app, helping them to share their profile with their family. However, some users criticized the synchronization speed of connecting different devices, as they would expect to have parallel and same-time synchronization. It is noteworthy to mention that for the enhancement of the FeverApp, user experiences and subsequently the research project, users’ comments have been taken into consideration. For instance, the export of fever phases (i.e., entries) to PDF format as a feature was added to the app. The date and time entries as well as the search function in the Info Library have also been optimized.

Based on the quantitative analysis, users gave a review mostly on the first month of using the app. This highlights the importance of the first impressions of mHealth apps in the early days after the download. The majority of reviews were from intensive users, indicating their experience working with the app, hence valuable comments. The majority of the comments were from mothers in age group of 31–40 years with a high level of education. These findings confirm that younger individuals with higher education tend more to use mHealth apps.^35^ In concordance with previous analyses,^13^ we observed that the comments regarding the documentation process are slightly more frequent compared to the comments on the information function of the app. This could be due to the fact that the documentation feature of the app is the main interaction of the users with the app.

This study has its strengths and limitations. The current study displays the features that the users of an educative research based mHealth app has found helpful and highlighting the limitations that could cause low engagement of the users. Such information could be used for other mHealth apps, being research-based or general. Having the feedback feature within the app reduced the limitations reported by previous studies focusing on public reviews. This includes limitations in access to demographic information or length and period of using the app.^15,26^ The possibility of giving feedback within the app helps to evaluate users’ experiences and reviews based on their data entries and demographic information. Even though the public feedbacks in App Store and Google Play provide insightful knowledge for the improvement of mHealth apps, having the feedback option within the apps could shed more light on each app and consequently for mHealth apps in specific health fields.

Even though the FeverApp users are from different states in Germany, providing a good representation, only 22% of the app users answered the feedback questionnaire, thus, generalizing the reported data has limitations. Additionally, the intention of writing a review can be influenced by users’ situations or behaviours.^36^

## Conclusion

Users would like to use mHealth apps that have good designs, in addition to reliable information. It is specifically important that these apps are easy to use, saving users time instead of challenging them to understand their features. Since tutorials are not always used or remembered, intuitive functionality is a high priority. Privacy is a great concern of the users that can be addressed through several means such as ways of their distribution. For example, via the apps, users would like to have direct contact with their physicians or be able to share their data with them. Such features could not only provide trust but also a possible enhancement in sustainable use of the apps. To conclude, the perspectives of mHealth app users should be considered as a valuable source of information as well as a key factor of sustainable use. Accordingly, steps should be taken based on their reviews to improve each mHealth app individually.
